# Age-related changes in the susceptibility to visual illusions of size

**DOI:** 10.1038/s41598-024-65405-6

**Published:** 2024-06-25

**Authors:** Yarden Mazuz, Yoav Kessler, Tzvi Ganel

**Affiliations:** Department of Psychology, Ben-Gurion University of the Negev, 8410500 Beer-Sheva, Israel

**Keywords:** Visual perception, Aging, Visual illusions, Size perception, Human behaviour, Cognitive ageing

## Abstract

As the global population ages, understanding of the effect of aging on visual perception is of growing importance. This study investigates age-related changes in adulthood along size perception through the lens of three visual illusions: the Ponzo, Ebbinghaus, and Height-width illusions. Utilizing the Bayesian conceptualization of the aging brain, which posits increased reliance on prior knowledge with age, we explored potential differences in the susceptibility to visual illusions across different age groups in adults (ages 20–85 years). To this end, we used the BTPI (Ben-Gurion University Test for Perceptual Illusions), an online validated battery of visual illusions developed in our lab. The findings revealed distinct patterns of age-related changes for each of the illusions, challenging the idea of a generalized increase in reliance on prior knowledge with age. Specifically, we observed a systematic reduction in susceptibility to the Ebbinghaus illusion with age, while susceptibility to the Height-width illusion increased with age. As for the Ponzo illusion, there were no significant changes with age. These results underscore the complexity of age-related changes in visual perception and converge with previous findings to support the idea that different visual illusions of size are mediated by distinct perceptual mechanisms.

## Introduction

As the population continues to age, the impact of aging on cognition and visual perception has become an increasingly pertinent and multifaceted area of study, with implications for both individual well-being and societal adaptation^1^. Over the past decades, researchers have documented empirical findings that shed light on the age-related changes in perceptual abilities. These changes involve a decline in various aspects of visual perception, including face perception^2,3^, contrast sensitivity at medium and high frequencies^4–6^, perception of orientation^7,8^, color perception^9^, some aspects of motion perception^10,11^ and more (for reviews, see Refs.^12,13^).

Theoretical frameworks grounded in the Bayesian model propose that perception is a result of integrating two key components: (1) an individual's prior knowledge of the world, and (2) sensory input. The influence of these factors is contingent upon the level of ambiguity in the sensory input. As sensory input becomes increasingly noisy, perception relies more heavily on prior knowledge^14–16^. Applying this logic to the aging population, it has been suggested that the strengthened prior knowledge acquired throughout the lifespan of older adults, coupled with the heightened noise in sensory input due to ocular and neural changes, leads to increased perceptual reliance on prior knowledge with ageing^17–19^.

An effective method for probing ageing effects on perception and for investigating hypotheses derived from the perceptual Bayesian model is through the examination of perceptual illusions. Perceptual illusions occur when predictive processes take precedence over sensory evidence, and the magnitude of illusions offers insights into the relative reliance on prior knowledge^14,15,20^. Extending this rationale to explore aging effects, Dowlati et al.^21^ employed a visual illusion paradigm of bistable perception, where participants viewed an ambiguous visual stimulus that could be perceived in two different ways, across diverse age groups, that included young and older adults. The authors incorporated a manipulation aimed to bias participants' priors. Specifically, the researchers presented participants with a cue prior to a bistable stimulus that was intended to bias their initial interpretation of the ambiguous figure. The behavioral results showed that the young adults group exhibited a strong bias in response to the manipulation compared to the older adults. In other words, the younger participants were more influenced by the prior cue and tended to perceive the bistable stimulus in the direction suggested by the cue, whereas the older adults were less affected by the cue and maintained more stable perceptual interpretations. Furthermore, their findings indicate that older adults exhibit increased activation in anterior brain regions compared to their younger counterparts, suggesting heightened reliance on top-down connections when processing ambiguous stimuli.

Another study by Chan et al.^17^, again grounded in the Bayesian model framework, provides compelling evidence for increased reliance of the aging brain on perceptual priors. This was demonstrated through the utilization of the sound-induced flash illusion, concurrently recorded with magnetoencephalography (MEG). Notably, the findings revealed that older adults exhibited greater susceptibility to the illusion compared to the younger group. Furthermore, the study observed increased pre-stimulus beta-band activity in older compared to younger adults, aligning with predictions derived from Bayesian microcircuit theories. These theories propose a link between priors and predictions with beta-band activity, thus providing empirical support for the intricate relationship between aging, perceptual priors, and neural oscillations.

Although there are supportive findings for the hypothesis of increased reliance on priors with age, it is crucial to recognize that such a proposition may be overgeneralized and might not accurately describe the complexity of the aging brain. Existing literature has established an absence of a common factor for individual differences in vision, particularly regarding the mechanisms that underlie visual illusions^22–25^. The recognition that different illusions are mediated by distinct perceptual mechanisms raises the possibility that aging may exert differential effects on these diverse mechanisms.

To investigate this idea, Trewartha and Flanagan^26^ looked at possible disparities between young and older participants in perceiving and manipulating objects within the context of the size- and material-weight illusion. The size-weight illusion manifests as the perception of smaller-sized objects (of heavier materials) as heavier than larger-sized objects (of lighter materials) of a similar weight, arising from an implicit assumption that objects made of heavier material are generally heavier. Notably, older and younger adults exhibited no differences in the magnitude of their initial size and material-weight illusions during a baseline assessment. Additionally, there were no observed differences in their ability to update their weight judgments.

Moreover, Grzeckowski et al.^24^ conducted a study with participants between the ages of 6–80 years old, who were tested in their susceptibility to 6 types of visual illusion: the Ebbinghaus, Müller-Lyer, simultaneous contrast illusion, Ponzo "hallway," White’s illusion, and the tilt illusion. Their findings indicated absence of significant correlations between age and the Müller-Lyer, simultaneous contrast illusion, and White’s illusion. However, in the case of the Ebbinghaus, Ponzo "hallway," and tilt illusion, a negative correlation emerged, suggesting a decline in the strength of these illusions with age. We note that this decline resulted primarily from larger susceptibility to the illusions in children rather than a decrease in susceptibility during adulthood. Within the adult group (age range 18–80), there appeared to be a tendency towards a negative association between age and between the Ebbinghaus and tilt illusions, albeit statistically insignificant. Such a relationship was not observed for the Ponzo illusion. This study underscores the necessity of recognizing the diverse impact of aging on distinct perceptual mechanisms associated with visual illusions. These findings also imply potential variations in susceptibility to different visual illusions among adults across different age groups, thereby emphasizing the intricate nature of perceptual processes throughout the lifespan.

In the current study, we aimed to investigate the effect of ageing on perceptual mechanisms that mediate three basic illusions of size. We base our assumptions on a Bayesian conceptualization of the aging brain, which posits a generalized increase in the weighting of priors over sensory information for the older adult population. Our objective is to explore and identify potential variations in the impact of age on different functions and mechanisms, introducing a nuanced perspective that acknowledges the possibility of diverse patterns of influence. To explore this idea, we employed a standardized test of the susceptibility to visual illusions, recently developed in our lab, the Ben-Gurion University Test for Perceptual Illusions (BTPI^25^). The BTPI is an online battery designed to measure individual differences in susceptibility to visual illusions. It showed high reliability in its measurements of the three prominent size illusions included in the battery: the Ebbinghaus, the Ponzo, and the Height-width illusions. The BTPI also measures perceptual resolution to size, which is reflected by the just noticeable difference (JND) in detecting size differences within each of the illusions (The battery can be freely accessed through the following link: https://app.gorilla.sc/openmaterials/514763).

## Methods

### Participants

Participants from three different age groups (young: 20–39 years, N = 80; middle-aged: 41–59, N = 70; old adults: 61–85, N = 80) were recruited through the Prolific platform, based on their age. Participants in all age groups had a record of successful participation in previous experiments in Prolific (100% successful completion of previous experiments). The experimental session lasted approximately 20 min, and participants were compensated with 3.13 euros. In line with prior research^25^, only participants exhibiting a goodness of fit (GOF) score exceeding 0.7 in all illusion tasks were included in the final analysis. One participant from the young group was excluded as he did not respond to more than 50% of the trials in each illusion. In total, the analysis included a sample of 229 participants, with 79 participants in the young adult group (age: M = 28.1, SD = 5.74), 70 participants in the middle-aged adults’ group (age: M = 48.3, SD = 5.15), and 80 participants in the old adults’ group (age: M = 67.4, SD = 4.71). Given that the old adult group has the smallest representation in Prolific’s population, and to make sure that a similar demographic profile across all age groups, we first ran the experiment on the old adults group and then recruited the participants from the other groups based on their demographics. In particular, many of the participants (34 out of 80) in the old adults groups were from the UK, so we recruited a similar number of participants from the UK for the other age groups as well (31 in the young adult group, 32 in the middle-aged adult group). Other demographic aspects of the age groups (country of residence, gender) were similar between the groups. All participants signed an informed consent form prior to beginning the experiment. The experimental protocol was approved by the ethics committee of the Department of Psychology in Ben-Gurion University of the Negev. The study adhered to the ethical standards of the Declaration of Helsinki.

### Procedure

**Figure 1:**
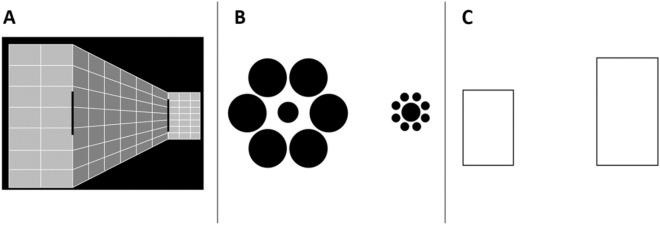
The stimuli used in the BTPI. (**A**) Ponzo illusion—the participants were instructed to choose the longer object. (**B**) Ebbinghaus illusion—the participants were instructed to choose the larger central circle. (**C**) Height–width illusion—the participants were instructed to choose the wider rectangle.

The participants performed the BTPI battery^25^. This battery uses an adapted version of the method of constant stimuli, comprising three visual illusions: Ponzo, Ebbinghaus, and the Height-width illusion (see Fig. 1). Each illusion was presented in a separate block, with a fixed order of Ponzo, Ebbinghaus, and then the Height–width illusion. Within each block, stimuli were randomly presented (12 repetitions of each of 12 standard-reference combinations, 144 trials overall). Participants indicated their choice for which object is bigger in each of the trials using the keys K and S on a standard keyboard to indicate the right and left object, respectively. In the Ponzo block, each trial began with a 1000 ms fixation cross, followed by a presentation of the stimuli. The stimuli remained on the screen until the participant's response was registered, with a maximum duration of 3000 ms. If no response was made within 3000 ms, the subsequent trial began automatically. For the Ebbinghaus and Height–width illusions, trials began with a 1000 ms fixation cross, followed by a 1000 ms presentation of stimuli, after which participants indicated their response. The BTPI's test–retest reliability is considered high for the illusions’ magnitude and moderate for the Just Noticeable Differences (JNDs, see Ref.^25^).

### Data analysis

Trials in which participants did not respond within the designated time limit (3000 ms) were excluded from the analysis. Overall, 0.08% trials were omitted from the young group’s data, 0.07% from the middle-aged group’s data, and 0.1% from the old adults group’s data. For each participant, we computed the proportion of trials in which they reported that the reference stimulus appeared larger (or wider) than the standard. Subsequently, we fitted the data to a sigmoid function ($\frac{1}{1+{e}^{-\frac{x-A}{B}}}$). From this analysis, we extracted values for the Point of Subjective Equality (PSE), Constant Error (CE), JND, Reaction Time (RT), and the Goodness of Fit (GOF).

**Figure 2:**
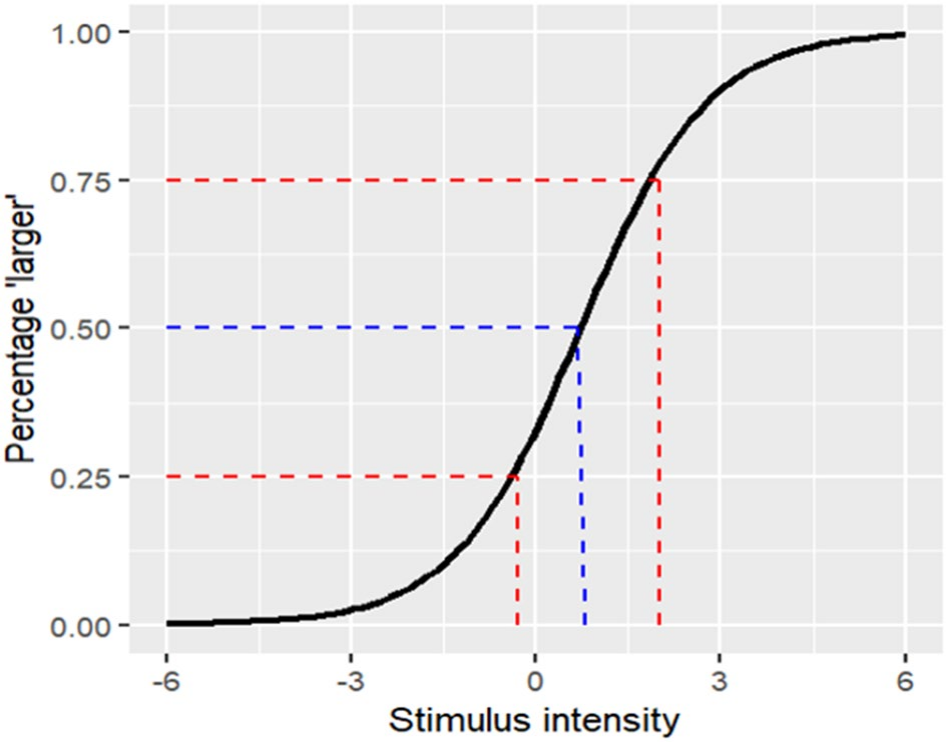
An illustration of the psychophysical curve. The x-axis represents the magnitude of the reference stimulus. The y-axis represents the percentage of trials by which the participant reported that the reference stimulus is larger than the standard stimulus, both embedded in the illusion. The black curve is the fitted sigmoid function that represents the participant’s data. The blue line marks the PSE, the value in which the participant perceived both stimuli as equal. The red lines represent the area of uncertainty, which equals two times the JND.

The CE signifies the magnitude of the illusion and was calculated by subtracting the value of the PSE (50% "larger" responses) from the value of the standard stimulus. The JND represents perceptual resolution for size differences within the context of the illusion and was computed by dividing the range between 25% and 75% of the function by two (see Fig. 2). To enhance clarity, raw scores for CEs and JNDs were transformed to percentage scores for each participant, compared to the standard stimulus. To remove outliers, participants with JND values greater than 3 standard deviations from the illusion-specific average were excluded from the analysis.

Reaction times (RTs) were recorded for each trial, and the mean RT was calculated for each participant in each illusion. To remove outliers, we excluded trials in which the RT exceeded 3 standard deviations from the participant's average RT within each illusion. Note, that the RT for the Ponzo illusion task was computed from the presentation of the stimuli until the participants' responses. However, for the Ebbinghaus and Height–width illusions, RT measurement commenced when participants were presented with the response display (following the 1000 ms exposure to the stimuli) in which the participants were required to indicate their response. Lastly, the GOF (goodness of fit) was computed as the squared correlation coefficient between the observed values and the values predicted by the sigmoid model. The GOF provides a numerical measure of the model's ability to accurately represent the underlying pattern of results.

For each illusion, we conducted three One-Way Analyses of Variance (ANOVAs) to examine statistical differences in CE, JND, and RT among young, middle-aged, and older adults’ groups.

## Results

Tables 1 and 2 display the average CEs and JNDs in each illusion for each age group. The differences in sample sizes between the illusions are due to the exclusion of participants with goodness-of-fit (GOF) values below 0.7 or participants with JND values greater than 3 standard deviations from the illusion-specific average.

**Table 1:** The CEs for each illusion for each age group (in percentages). The CE represents the magnitude of the illusion.

Illusion	Group	N	Mean	SD	Range		Skewness		Kurtosis	
					Minimum	Maximum	Skewness	SE	Kurtosis	SE
Ponzo	Young	60	44.38	22.93	3.52	98.50	0.42	0.31	− 0.27	0.61
	Middle aged	53	39.54	23.00	− 16.65	106.50	0.73	0.33	1.83	0.64
	Old	65	45.10	21.50	12.91	95.80	0.62	0.30	− 0.42	0.59
Ebbinghaus	Young	64	48.20	14.25	12.57	81.80	− 0.11	0.30	− 0.27	0.59
	Middle aged	63	41.44	12.33	16.74	69.80	0.27	0.30	− 0.59	0.60
	Old	76	36.11	9.91	14.92	57.40	0.06	0.28	− 0.56	0.55
Height–width	Young	71	1.94	4.22	− 10.71	11.60	− 0.44	0.29	0.73	0.56
	Middle aged	68	3.74	2.82	− 3.70	12.40	0.17	0.29	1.62	0.57
	Old	78	5.14	2.74	− 1.51	12.90	0.37	0.27	0.20	0.54

**Table 2:** The JNDs for each illusion for each age group (in percentages). The JND represents visual resolution for differences in size.

Illusion	Group	N	Mean	SD	Range		Skewness		Kurtosis	
					Minimum	Maximum	Skewness	SE	Kurtosis	SE
Ponzo	Young	64	7.26	2.87	1.76	13.95	0.15	0.30	− 0.52	0.59
	Middle	63	6.13	2.56	0.40	14.64	0.45	0.30	1.06	0.60
	Old	76	6.56	3.05	0.39	15.76	0.87	0.28	0.90	0.55
Ebbinghaus	Young	71	4.39	2.05	1.34	12.67	1.43	0.29	2.88	0.56
	Middle	68	3.17	1.27	1.10	6.47	0.52	0.29	− 0.12	0.57
	Old	78	3.40	1.66	0.24	8.85	0.65	0.27	1.14	0.54
Height–width	Young	60	10.41	4.90	2.91	23.21	0.81	0.31	0.32	0.61
	Middle	53	9.59	5.20	2.36	25.11	1.26	0.33	1.30	0.64
	Old	65	10.88	6.11	1.73	25.58	0.52	0.30	− 0.86	0.59

**Figure 3:**
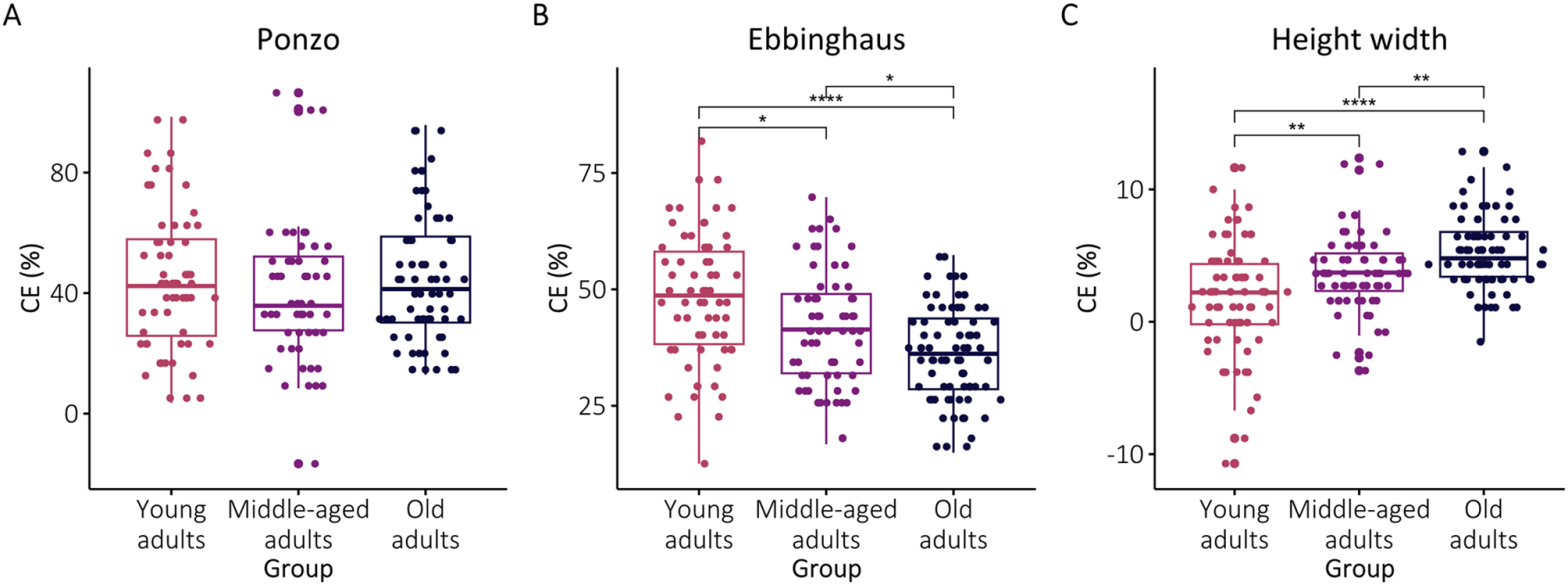
CEs (the magnitudes of the illusion, in percentage) for the Ponzo, Ebbinghaus and Height-width illusions (from left to right, respectively). The box plots represent 50% of the central data (IQR), and the insert lines represent the medians. The whiskers show the range of the data, they extend to 1.5 times the IQR from Q1 and Q3. Individual data points are also presented for each category. *p < 0.05, **p < 0.01, ***p < 0.001.

The statistical results of the one-way ANOVA are presented in Table 3. The descriptive results for the CE measure are presented in Fig. 3, and post-hoc comparisons are presented in Table 4. As can be seen in Fig. 3, the magnitude of the Ebbinghaus illusion significantly decreased with age. Post-hoc comparisons using the Tukey test indicated that younger adults exhibited significantly higher CEs than middle-aged and older adults. Furthermore, middle-aged adults showed higher CEs than old adults. This indicates a systematic reduction in susceptibility to the Ebbinghaus illusion with age. In sharp contrast, the susceptibility to the Height-width increased with age. The one-way ANOVA for the Height-width illusion showed a significant effect of age group but in a direction opposite to the one found for the Ebbinghaus illusion. Post-hoc comparisons showed that young adults exhibited lower susceptibility to the illusion compared to middle-aged adults and older adults. Additionally, middle-aged adults were less susceptible than old adults. Notably, unlike the Ebbinghaus and Height-width illusions, there were no significant differences between the age groups along the susceptibility to the Ponzo illusion.

**Table 3:** The results of the one-way ANOVAs for the CE, JND, and RT in each illusion.

Measure	Illusion	F	df1	df2	p-value	η^2^
CE	Ponzo	1.02	2	175	0.362	0.011
	Ebbinghaus	17.18	2	200	< 0.001	0.147
	Height–width	17.37	2	214	< 0.001	0.140
JND	Ponzo	0.82	2	175	0.440	0.001
	Ebbinghaus	2.54	2	200	0.081	0.025
	Height–width	10.32	2	214	< 0.001	0.088
RT	Ponzo	15.25	2	175	< 0.001	0.148
	Ebbinghaus	9.37	2	200	< 0.001	0.086
	Height–width	24.62	2	214	< 0.001	0.187

**Table 4:** Post hoc comparisons for CEs. *p < 0.05, **p < 0.01, ***p < 0.001.

Illusion	(I) Age group	(J) Age group	Mean difference	p-value
Ponzo	Young Young	Middle	4.84	0.488
		Old	− 0.72	0.983
	Middle	Old	− 5.56	0.376
Ebbinghaus	Young Young	Middle	6.76**	0.006
		Old	12.09***	< 0.001
	Middle	Old	5.33*	0.029
Height–width	Young Young	Middle	− 1.79**	0.005
		Old	− 3.2***	< 0.001
	Middle	Old	− 1.41*	0.030

We note that the different presentation method (3 s presentation duration) of the Ponzo display could potentially introduce differences along its susceptibility with age. We therefore included an additional analysis that shows that the general pattern of responses does not change even when presentation times are equated between age groups (see Supplementary materials s1).

**Figure 4:**
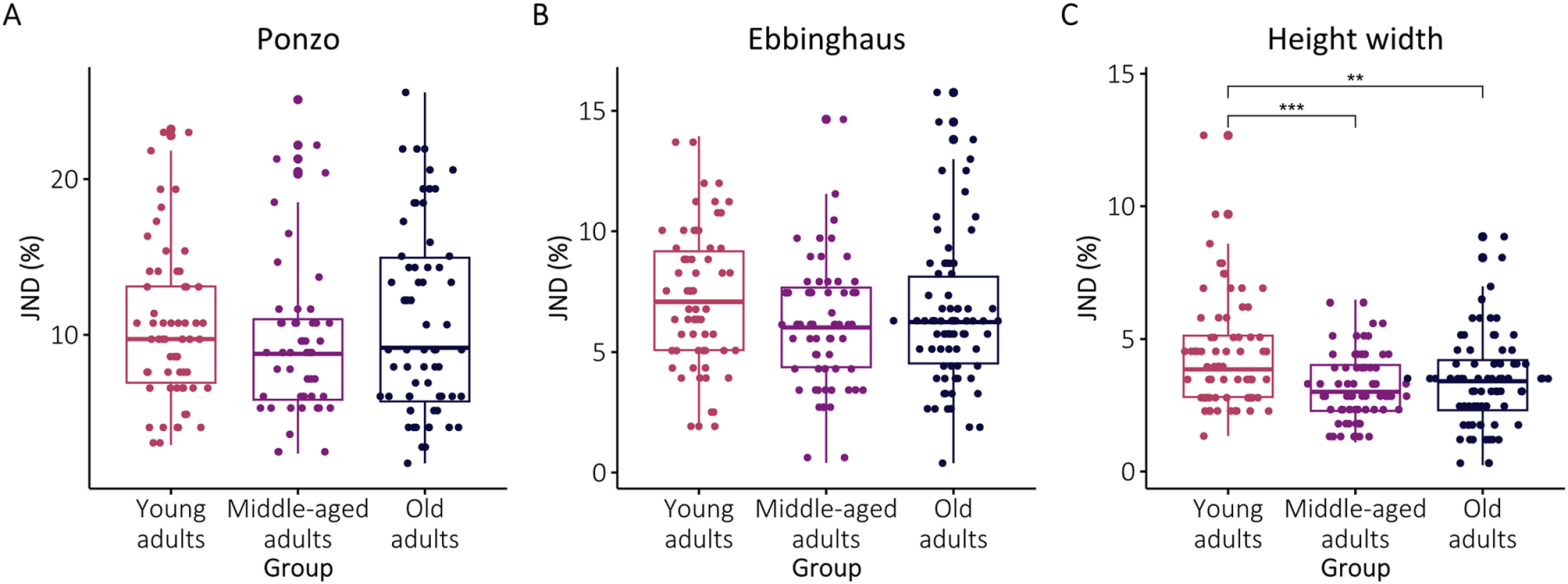
JNDs (in percentage) for the Ponzo, Ebbinghaus and Height–width illusions (from left to right, respectively). The box represents 50% of the central data (IQR), the insert lines represent the median. The whiskers show the range of the data, they extend to 1.5 times the IQR from Q1 and Q3. Individual data points are also presented for each category. *p < 0.05, **p < 0.01, ***p < 0.001.

The ANOVA analysis for the JNDs revealed that age exhibited an effect on the resolution to size differences only for the Height–width illusion (Table 3). Post-hoc comparisons showed that young adults exhibited lower sensitivity to size differences (larger JNDs) than middle-aged and older adults. For the Ebbinghaus and Ponzo illusions, age did not exert a significant influence on JNDs (see Fig. 4 and Table 5).

**Table 5:** The results of the post-hoc comparisons for JNDs. *p < 0.05, **p < 0.01, ***p < 0.001.

Illusion	(I) Age group	(J) Age group	Mean difference	p-value
Ponzo	Young	Middle	0.82	0.705
	Young	Old	− 0.47	0.880
	Middle	Old	− 1.30	0.410
Ebbinghaus	Young	Middle	1.13	0.07
	Young	Old	0.69	0.323
	Middle	Old	− 0.43	0.646
Height–width	Young	Middle	1.22***	< 0.001
	Young	Old	0.99**	0.001
	Middle	Old	-0.23	0.697

**Figure 5:**
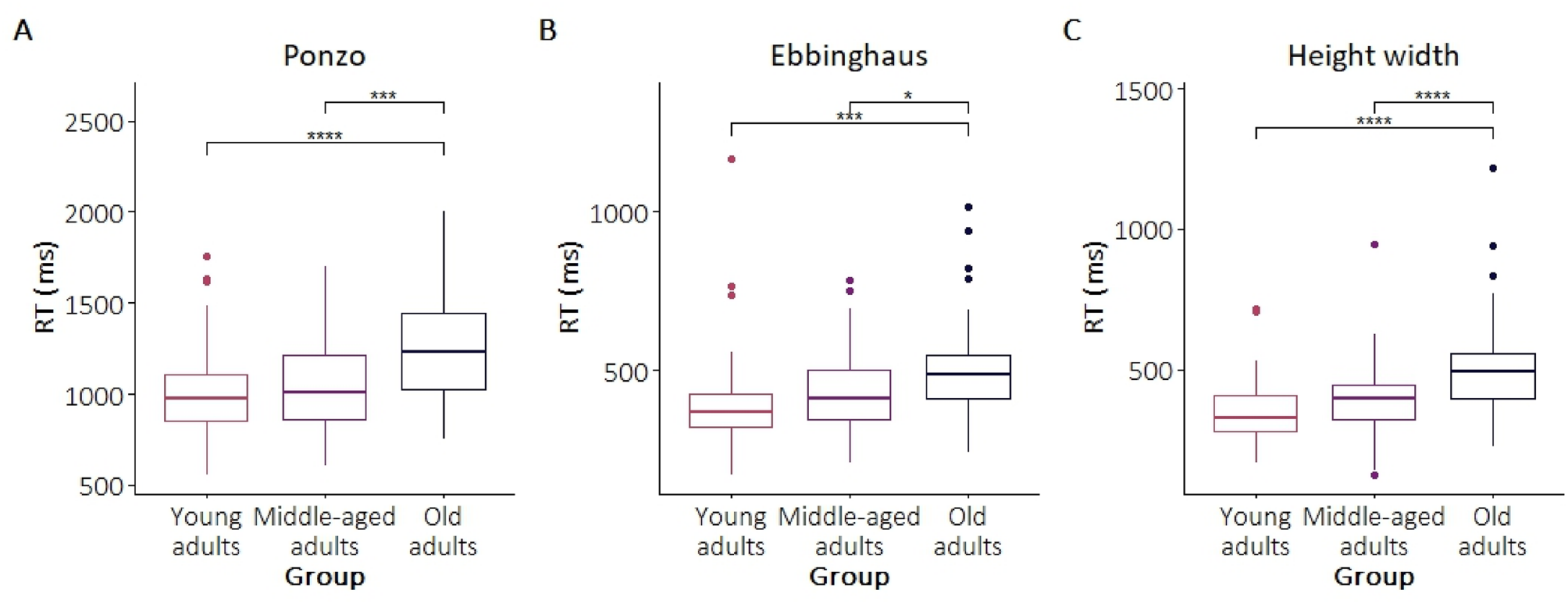
Reaction times for the Ponzo, Ebbinghaus and Height-width illusions (from left to right, respectively). The box represents 50% of the central data (IQR), and the insert lines represent the median. The whiskers show the range of the data, they extend to 1.5 times the IQR from Q1 and Q3. The data points beyond this range are outliers and are plotted as points. *p < 0.05, **p < 0.01, ***p < 0.001.

As anticipated due to the general slowing effect with ageing, an effect of age was evident in the reaction times across all illusions (Table 3). Subsequent Tukey post-hoc test showed that older adults had slower reaction times than both young and middle-aged adults (Fig. 5 and Table 6). To supplement the current analysis, we also analyzed the data with age as a continuous variable. The results were generally in line with the main analysis (see Supplementary materials s2).

**Table 6:** The results of the post-hoc comparisons for RTs. *p < 0.05, **p < 0.01, ***p < 0.001.

Illusion	(I) Age group	(J) Age group	Mean difference	p-value
Ponzo	Young	Middle	− 50.3	0.576
	Young	Old	− 249***	< 0.001
	Middle	Old	− 198***	< 0.001
Ebbinghaus	Young	Middle	− 31.2	0.408
	Young	Old	− 97.9***	< 0.001
	Middle	Old	− 66.7*	0.013
Height–width	Young	Middle	− 50.5*	0.070
	Young	Old	− 151***	< 0.001
	Middle	Old	− 100***	< 0.001

## Discussion

This study investigated the effects of age on adults’ susceptibility to three prominent size illusions: the Ponzo, Ebbinghaus, and Height–width illusion. The results revealed distinct patterns of age-related changes for each of the illusions. For the Ebbinghaus illusion, there was a systematic reduction in the illusion’s magnitude with age; older adults were significantly less susceptible to this size-contrast illusion compared to young and middle-aged adults. In sharp contrast, the Height-width illusion, which is driven by the holistic processing of shape, exhibited an opposite trend, with older adults showing greater susceptibility to the illusion relative to their younger counterparts. For the third illusion of size, the Ponzo illusion, triggered by the influence of 2D depth cues, there were no significant effects of age. Furthermore, for all illusions, the analysis of reaction times showed the expected general slowing effect with ageing. These findings challenge a general notion of an overall increase in reliance on prior knowledge with age, as proposed by Bayesian theories of perception. Instead, our results indicate that the impact of aging on visual perception may be more nuanced, with varying effects across different perceptual domains and illusions.

To interpret our results more closely within a Bayesian framework, we suggest that the illusion’s magnitude, namely the CE, can be associated with the participants’ priors, reflecting the perceptual mechanisms that trigger the illusion. On the other hand, JNDs (Just Noticeable Difference), which reflect the resolution for differences in size, are more closely related to the participants’ sensory input^27^. The present findings primarily indicated age-related changes in the priors of different illusions. Priors related to size-contrast effects, which mediate the Ebbinghaus illusion, seem to diminish with age. Conversely, priors concerning global, or configural perception of shape, appear to increase with age, as observed in the case of Height–width illusion. For the Ponzo illusion, for which priors reflect size constancy mechanisms, there were no significant changes with age. Unlike CEs, JNDs in the current study largely remained stable, and did not increase with age for any of the illusions. This pattern of results for ageing cannot be easily accommodated by the Bayesian framework that posits a general decline in the reliability of sensory input in older adults. It is possible therefore, that despite the fact that some perceptual visual abilities decline with age^12,13^, size discrimination abilities remain intact.

Grzeckowski et al.^24^ investigated ageing effects on the susceptibility to different perceptual illusions, among them the Ponzo "hallway" and the Ebbinghaus illusions, across a range of ages from 6 to 86 years. Their findings revealed a decline with age along the perceptual magnitude of both the Ponzo and Ebbinghaus illusions. While our study bears similarities to that of Grzeckowski et al., it is important to note that most of the ageing effects in Grzeckowski et al.’s study were attributed to differences between children and adults in all ages. Consequently, when specifically comparing the magnitude of illusions between the young and middle-aged adults (ranging from 18 to 60 years) and between older adults (individuals aged above 60) in their study, it appears that there persisted a visible decline in the strength of the Ebbinghaus illusion, while no noticeable impact of age was found on the susceptibility to the Ponzo illusion. However, probably due to the smaller sample of adults and old adults in Grzeckowski et al., there were no significant differences along the magnitude of the illusions in adulthood. Yet, the findings of Grzeckowski et al. are generally in line with the findings of the current study, showing that different visual illusions are differentially affected by ageing.

In a relevant study that looked at ageing effects on the Ebbinghaus illusion, Coren and Porac^28^ measured the illusion’s magnitude in 688 participants in ages of 5–70. Unlike the design used in the current study or that used in Grzeckowski et al.’s^24^ study, Coren and Porac used two separate displays of the Ebbinghaus illusion, one for the overestimation portion (the target circle surrounded by the smaller distractors) and a separate one for the underestimation portion of the illusion (the target circle surrounded by the larger distractors). The effects of ageing were non-linear and were different for the two illusory displays; for the overestimation portion, there was a slight decrease in the illusion’s magnitude between young and middle-aged adulthood, after which the illusion’s magnitude fluctuated around zero until the age of 70 years. For the underestimating portion of the illusion, there was a small increase in the illusion’s magnitude from young to middle-aged adulthood, with a hint of reversal (decrease in the illusion’s magnitude) between the ages of 60 and 70 years. This pattern of results is different from the one found in the current study, in which the magnitude of the illusion gradually decreased with age (between 20 and 80 years). Yet, this discrepancy can be attributed to substantial differences in the nature of the illusory displays used in the two studies. The “separated” versions of the illusion used by Coren and Porac are substantially different from the “combined” version used here and in other recent studies. First, the illusory effects of the separated version are significantly smaller (about 4.5% for the underestimating portion; less than 1% for the overestimation portion) than the illusory effects of the combined version (around 42% in the present study). Therefore, it can be argued that presenting the two portions of the illusion within a single display does not result in additive effects of its two parts. Rather, the size-contrast contextual effects in the combined version of the illusion are also qualitatively different from the effects of the two separate parts of the illusion. These results extend the results of the present study in that they show that ageing not only has different effects on different visual illusions, but it also could have different effects for particular variants of size-contrast illusions.

For the Ponzo illusion, our findings indicate no significant age-related variations in the susceptibility to the illusion among adults. This result is not in line with the results of a previous study that looked at the effects of ageing on the susceptibility to the Ponzo illusion. In particular, Leibowitz and Judisch^29^ noted an increase in the magnitude of the illusion from early childhood to adolescence, followed by a period of stability until the age of 50, and then a decline in the susceptibility the illusion in older adults. The discrepancy between our findings and those of Leibowitz and Judisch may be attributed to differences in the visual stimuli used across the studies. While Leibowitz and Judisch’s design employed simple line drawings, our study utilized visual displays with richer depth cues. In a recent review, Yildiz et al.^30^ suggested that variations in the specific attributes of the Ponzo illusion can lead to mixed results across studies. In a related study, Cretenoud et al.^31^ explored the influence of context on the Müller-Lyer and Ponzo illusions and their relations with age. The results showed a slight reduction in the illusion with age, without any significant interaction between age and context. Nevertheless, disparities between their methodology and ours are evident. Primarily, their sample predominantly consisted of individuals under the age of 23, thus limiting the generality of their results to older adults. Additionally, Cretenoud et al. employed an indirect comparison task, contrasting with our direct comparison method where two objects were integrated in the illusion context. In general, given the impact of ageing on the Ponzo illusion was relatively understudied, the current research helps to fill this gap and showed consistency of the influence of the illusion throughout adulthood and adult age using an established tool and for a large sample and participant age range^25^. The current pattern of results is also in line with the results of a recent study that looked at the effect of ageing on visual size perception in non-illusory displays^32^.

Norman et al.^32^ compared younger and older adults in their ability to judge visual object size under different viewing conditions, utilizing depth cues of linear perspective. The results showed no significant difference in accuracy or precision between the younger and older adult groups. Although Norman et al. focused on size perception of real, physical objects, with no illusory background, while the present study involves illusory, two-dimensional stimuli, Norman et al.’s results are in general agreement with the current findings of the patterns of JNDs and CEs in the case of the Ponzo illusion. The idea that size constancy mechanisms remain intact in older ages, as Norman et al.’s results and the present data suggest, implies that priors and likelihoods regarding size and distance perception remain intact with age. It is important to note that differences in the CE and JNDs might indicate changes in priors and in sensory inputs, but age-related changes could also reflect more complex changes in the weighting between priors and input, which could not be fully captured by the present experimental settings. This nuanced interpretation underscores the complex interplay between sensory input and prior knowledge in shaping perceptual experience. To further explore such age-related changes the present investigation can be extended in future research by a direct comparison between CEs and JNDs in illusory and non-illusory displays. This could provide further insights into how size constancy and size perception develop with age.

The observed decrease along the magnitude of the Ebbinghaus illusion with age can be attributed to changes in the underlying perceptual mechanisms. Previous research has suggested that both the ability to integrate local contour elements into a coherent global shape, as well as the ability to perceptually group those local elements together, decline with age^33–36^. The Ebbinghaus illusion is primarily driven by the way the local surrounding elements are processed as context to the primary target. However, as people age, decreased sensitivity in contour integration, which links the local edge segments into a unified global percept, can diminish the saliency of the surrounding elements^33^. In addition, the age-related decline in perceptual grouping ability can also contribute to the reduced influence of the surrounding elements on the perceived size of central objects^37^. As individuals have more difficulty grouping the local elements into a coherent global shape, the contextual influence of the surrounding objects on the central target can be diminished.

Although grouping and contour integration are perceptual abilities that require some aspect of global processing, our findings show an increased susceptibility to the Height-width illusion with ageing. The Ebbinghaus and the Height-width illusions are both considered size-contrast illusions. Yet, the Height-width illusion emerges from holistic processing within elements that belong to the same object^38^, as opposed to the Ebbinghaus illusion, which is a between-object illusion. This indicates that there may be differences in the effects of ageing on different shape processing mechanisms (for a review, see Ref.^39^).

While much of the existing literature that looked at age-related changes in global shape processing has focused on contour integration tasks, where observers identify contours, detect contrasting elements within a contour or recognize contours embedded in noise, there is a noticeable lack of studies focusing on holistic processing of shape and on global perception within objects. Meng et al.^40^ examined age-related differences in global topological and local geometrical visual perception using the Configural Superiority effect. Global topological properties refer to the fundamental connectivity and continuity of visual closed shapes (shapes with holes), which remain invariant under smooth deformations. Local geometrical properties, on the other hand, involve more specific shape features such as orientation, parallelism, and collinearity. The results showed that older adults exhibited higher Inverse Efficiency Scores (IEs) for local geometrical discrimination tasks compared to younger adults, but no differences were found for global topological discrimination tasks. This suggests that global topological perception remains relatively intact with age. The current findings, however, suggest increased reliance on this mechanism with age. Therefore, more research is required to better understand the effects of age on different aspects of shape perception.

In addition to examining susceptibility to visual illusions, we also investigated participants' JND for the different illusions. The results show no ageing effects on JNDs for the Ebbinghaus and the Ponzo illusions. Surprisingly, however, for the Height–width illusion, young adults had higher JNDs compared to middle-aged and older adults. This finding suggests that while older adults may be more susceptible to the illusion itself, they demonstrate greater precision in discriminating size differences within the illusory context. Given the limited research on this aspect of visual perception and the unexpected nature of our results, these findings raise questions as for the relations between JNDs and PSEs in the context of illusions, and the effect of ageing on these relations.

In summary, the results of this study reveal differential effects of ageing on the susceptibility to different visual illusions of size. This indicates that age has different effects on perceptual mechanisms even within a single domain of size perception, aligning with previous studies that suggested a lack of a common mechanism for size perception in vision. These findings also emphasize the complexity of age-related changes in visual perception and underscore the need for further elucidation of the underlying mechanisms of visual perception with ageing.

### Supplementary Information

Supplementary Information 1.

Supplementary Information 2.

## Data Availability

An online version of the BTPI as well as an online demo version of the BTPI are accessible at https://app.gorilla.sc/openmaterials/514763. The raw data, preprocessed data, and R code used for analysis are available on the Open Science Framework (OSF) repository at https://osf.io/2ryxj/.
