# Supplementary material for: Age-related changes in the susceptibility to visual illusions of size
Source: Sci Rep. 2024 Jun 25;14:14583. doi: 10.1038/s41598-024-65405-6 (PMC11199550; doi:10.1038/s41598-024-65405-6)
Supplement: Supplementary file 1 — Supplementary Information 1. [file 41598_2024_65405_MOESM1_ESM.docx]

Controlling for potential age-related differences in the presentation times of the Ponzo illusion.

The longer presentation times and the different presentation method for the Ponzo illusion, compared to the Ebbinghaus and Height-width illusions, are based on the way we originally developed the BTPI (Mazuz et al., 2023). However, in the context of the present study, it could raise the issue of whether older participants had sufficient time to process the background, potentially influencing the illusion's magnitude in this age group. In our original paper, the decision to use a version in which the Ponzo display is presented until the participants’ response was based on the larger reliability of the data when comparing this design to an alternative design in which presentation time was limited to 1000ms (as in the case of the other illusions). In addition, for the Ponzo illusion only, we found that reaction times and presentation times were not correlated to the illusion’s magnitude (Mazuz et al., 2023). This was the rationale for including the illusion in its present format in our final BTPI version. Since BTPI was primarily validated on younger participants, it is possible to argue that the interaction between age and exposure time could influence the illusion’s magnitude for older participants.

To address this issue, we tested whether equating exposure times between older and younger adults would change the pattern of results. For this purpose, we performed an additional analysis in which we included, for the older adult group, only trials in which reaction times were faster than 1500 ms. As can be seen in the tables below, exposure times in the new analysis are similar across the age groups. Still, there were no significant differences between younger and older participants in their susceptibility to the illusion (F(2,165) = 1.23, p = .294). Subsequent analyses showed no significant differences in the Ponzo CEs between the age groups (F(2.165, p = .357), aligning with the original results. This new analysis reinforces the idea that the present findings are not confounded by exposure times.

| Group Descriptives | | | | | | | | | | | |
| --- | --- | --- | --- | --- | --- | --- | --- | --- | --- | --- | --- |
|  | | **Group** | | **N** | | **Mean** | | **SD** | | **SE** | |
| RT Ponzo |  | Young |  | 60 |  | 1004.1 |  | 241.1 |  | 31.12 |  |
|  |  | Middle |  | 53 |  | 1064.5 |  | 255.3 |  | 35.06 |  |
|  |  | Old |  | 55 |  | 1014.8 |  | 123.2 |  | 16.61 |  |
| CE%_Ponzo |  | Young |  | 60 |  | 44.4 |  | 22.9 |  | 2.96 |  |
|  |  | Middle |  | 53 |  | 39.2 |  | 23.2 |  | 3.19 |  |
|  |  | Old |  | 55 |  | 44.3 |  | 17.8 |  | 2.40 |  |
|  | | | | | | | | | | | |

| One-Way ANOVA (Fisher's) | | | | | | | | | |
| --- | --- | --- | --- | --- | --- | --- | --- | --- | --- |
|  | | **F** | | **df1** | | **df2** | | **p** | |
| RT_Ponzo |  | 1.23 |  | 2 |  | 165 |  | 0.294 |  |
| CE%_Ponzo |  | 1.04 |  | 2 |  | 165 |  | 0.357 |  |
|  | | | | | | | | | |
