# Supplementary material for: Age-related changes in the susceptibility to visual illusions of size
Source: Sci Rep. 2024 Jun 25;14:14583. doi: 10.1038/s41598-024-65405-6 (PMC11199550; doi:10.1038/s41598-024-65405-6)
Supplement: Supplementary file 2 — Supplementary Information 2. [file 41598_2024_65405_MOESM2_ESM.docx]

We conducted further analysis on the data treating age as a continuous variable. The results aligned with the categorical analysis. Initially, we investigated the relationship between age and various variables to determine if it was linear or quadratic. The model comparison, detailed in Table 1s, favored the general linear model for the CE measure of the Ebbinghaus and Height-width illusions. This suggests that the magnitude of the Ebbinghaus illusion linearly decreases with age (𝑏𝐴𝑔𝑒 = -0.31, p < .001), while the magnitude of the Height-width illusion increases with age (𝑏𝐴𝑔𝑒 = 0.08, p < .001). Consistent with the ANOVA results, age did not predict the illusion magnitude of the Ponzo illusion (𝑏𝐴𝑔𝑒 = 0.07, p = .45).

Furthermore, linear regression models for Ponzo and Ebbinghaus indicated that age could not predict the Just Noticeable Differences (JNDs) for these illusions (Ponzo: 𝑏𝐴𝑔𝑒 = 0.01, p = .73; Ebbinghaus: 𝑏𝐴𝑔𝑒 = -0.02, p = 0.18). In contrast, the quadratic regression for Height-width revealed an initial decline in JND with increasing age (𝑏𝐴𝑔𝑒= -0.184, p < .001), followed by a subtle increase as age continued to rise (𝑏𝐴𝑔𝑒2= 0.002, p < .001). These results are consistent with our ANOVA findings and support our interpretations.

**Table 1s.** Model comparison. The bold values indicate the preferred model by BIC. The Bayes Factor (BF) was computed using the formula $BF= {exp}^{\frac{\Delta BIC}{2}}$, where ΔBIC is the difference in BIC values between the compared models. The package ‘bayestestR’ (Makowski et al., 2019) was utilized for the creation of the table and the calculation of the Bayes Factor.

| Measure | Illusion | Model | BIC (weights) | R2 | RMSE | Bayes factor |
| --- | --- | --- | --- | --- | --- | --- |
| CE | Ponzo | GLM | 1617.2 (0.326) | 0.003 | 22.34 | 2.26 |
|  |  | Quadratic | 1615.7 (0.674) | 0.023 | 22.12 |  |
|  | Ebbinghaus | **GLM** | **1598.8 (0.905)** | **0.165** | **11.94** | 9.55 |
|  |  | Quadratic | 1603.3 (0.095) | 0.169 | 11.92 |  |
|  | Height-Width | **GLM** | **1147.8 (0.935)** | **0.145** | **3.282** | 14.42 |
|  |  | Quadratic | 1153.1 (0.065) | 0.145 | 3.282 |  |
| JND | Ponzo | GLM | 1123.6 (0.479) | 7.81e-04 | 5.438 | 1.09 |
|  |  | Quadratic | 1123.4 (0.521) | 0.03 | 5.357 |  |
|  | Ebbinghaus | GLM | 1017.3 (0.605) | 0.009 | 2.85 | 1.53 |
|  |  | Quadratic | 1018.1 (0.395) | 0.03 | 2.819 |  |
|  | Height-Width | GLM | 866.3 (0.015) | 0.051 | 1.716 | 67.24 |
|  |  | **Quadratic** | **844.6 (0.997)** | **0.11** | **1.662** |  |
| **Table 2s.** Pearson correlations between the different measures of each illusion. CE and JND values are in percentages. Note. * p < .05, ** p < .01, *** p < .001 | | | | | | |

|  | **Age** | **CE Ebbinghaus** | **CE Height-width** | **CE Ponzo** | **JND Ebbinghaus** | **JND Height-width** | **JND Ponzo** | **RT Ebbinghaus** | **RT Height-width** | **RT Ponzo** |
| --- | --- | --- | --- | --- | --- | --- | --- | --- | --- | --- |
| **Age** | — |  |  |  |  |  |  |  |  |  |
| **CE Ebbinghaus** | -0.407*** | — |  |  |  |  |  |  |  |  |
| **CE Height-width** | 0.381*** | -0.02 | — |  |  |  |  |  |  |  |
| **CE Ponzo** | 0.057 | 0.293*** | 0.161* | — |  |  |  |  |  |  |
| **JND Ebbinghaus** | -0.095 | 0.46*** | 0.081 | 0.16* | — |  |  |  |  |  |
| **JND Height-width** | -0.227*** | 0.273*** | -0.129 | 0.174* | 0.363*** | — |  |  |  |  |
| **JND Ponzo** | 0.028 | 0.183* | 0.136 | 0.489*** | 0.175* | 0.164* | — |  |  |  |
| **RT Ebbinghaus** | 0.315*** | -0.205** | 0.06 | -0.025 | -0.018 | 0.099 | 0.043 | — |  |  |
| **RT Height-width** | 0.42*** | -0.177* | 0.149* | 0.045 | -0.064 | 0.075 | 0.022 | 0.739*** | — |  |
| **RT Ponzo** | 0.363*** | -0.165* | 0.215** | 0.118 | 0.028 | 0.05 | 0.163* | 0.279*** | 0.297*** | — |
|  | | | | | | | | | | |

We then proceeded to analyze Pearson correlations of the illusions with age as a continuous variable (Table 2s). The correlations between age and different measures were in line with our primary results, showing a negative correlation between the magnitude of the Ebbinghaus illusion and age, as well as between Height-width JND and age. Additionally, there was a positive correlation between the magnitude of the Height-width illusion magnitude and age. Correlations between age and RTs were found for all illusions.
